# A multi-professional survey of UK practice in the use of intra-articular corticosteroid injection for symptomatic first metatarsophalangeal joint osteoarthritis

**DOI:** 10.1186/s13047-023-00672-6

**Published:** 2023-10-17

**Authors:** Michael R. Backhouse, Jill Halstead, Edward Roddy, Vivek Dhukaram, Anna Chapman, Susanne Arnold, Julie Bruce

**Affiliations:** 1 Warwick Clinical Trials Unit, University of Warwick, Gibbet Hill, CV4 7AL UK; 2 University Hospitals Coventry & Warwickshire NHS Trust, Coventry, UK; 3 Leeds Community Healthcare NHS Trust, Leeds, UK; 4 School of Medicine, Primary Care Centre Versus Arthritis, Keele University, Staffordshire, UK; 5 Haywood Academic Rheumatology Centre, Haywood Hospital, Midlands Partnership University NHS Foundation Trust, Stoke-on-Trent, UK

**Keywords:** Metatarsophalangeal joint, Foot, Steroids, Osteoarthritis, Hallux rigidus, Surveys and questionnaires

## Abstract

**Background:**

The first metatarsophalangeal joint is the most common site of osteoarthritis (OA) in the foot and ankle. Intra-articular corticosteroid injections are widely used for this condition, but little is known about their use in practice. This study explored current practice within the UK National Health Service (NHS) relating to the administration of intra-articular corticosteroids for people with painful first metatarsophalangeal joint (MTPJ) OA.

**Methods:**

A cross-sectional survey using Qualtrics online survey platform (Qualtrics, Provo, UT, USA), distributed through professional bodies, special interest groups, and social media.

**Results:**

One hundred forty-four healthcare professionals responded, including podiatrists (53/144; 39%), orthopaedic surgeons (28/144; 19%), podiatric surgeons (26/144; 17%) and physiotherapists (24/144; 16%). Half of respondents administered up to 25 corticosteroid injections per year (67/136; 49%) but some administered more than fifty (21/136; 15%). Injections were administered across the healthcare system but were most common in hospital settings (64/136; 44%) followed by community (38/136; 26%), with less delivered in primary care (11/136; 8%). Half of respondents routinely used image-guidance, either ultrasound or x-ray/fluoroscopy (65/136; 48%) although over one third used none (52/136; 38%). Imaging guidance was more common amongst medical professionals (21/31; 68%) compared to non-medical health professionals (45/105; 43%).

Overall, methylprednisolone acetate was the most common corticosteroid used. Medical professionals mostly injected methylprednisolone acetate (*n* = 15/27; 56%) or triamcinolone acetonide (*n* = 11/27; 41%), whereas premixed methylprednisolone acetate with lidocaine hydrochloride was the most common preparation used by non-medical health professionals (41/85; 48%). When injecting non premixed steroid, lidocaine hydrochloride (15/35; 43%) was the most common choice of local anaesthetic for non-medical health professionals but medical professionals showed more variation between lidocaine hydrochloride (8/23; 35%) levobupivacaine hydrochloride (9/23; 39%) and bupivacaine hydrochloride (5/23; 22%).

**Conclusions:**

Multiple professional groups regularly administer intra-articular corticosteroids for symptomatic first MTPJ OA across a range of NHS healthcare settings. Overall, methylprednisolone acetate was the most commonly administered steroid and lidocaine hydrochloride the most common local anaesthetic. There was large variation in the use of imaging guidance, type and dose of steroid, local anaesthetic, and clinical pathways used in the intra-articular injection of corticosteroids for people with first MTPJ OA.

**Supplementary Information:**

The online version contains supplementary material available at 10.1186/s13047-023-00672-6.

## Background

Osteoarthritis (OA) represents a substantial and growing health burden for affected individuals and healthcare systems with broad socioeconomic costs [1, 2]. There is increasing evidence from population cohort studies that foot OA is common, but it has received less attention than other anatomical sites [3, 4]. This is despite growing evidence of the impact of foot OA on pain, physical activity, health-related quality of life, increased use of healthcare resources such as GP appointments, and potentially reduced life expectancy [3, 5–11].

The most commonly affected joint in the foot is the first metatarsophalangeal joint (MTPJ) [12]. Prevalence estimates vary between studies due to participant characteristics, variation in case definitions and methods for assessment. The prevalence of clinically diagnosed first MTPJ OA is unclear but estimates of population prevalence of radiographic OA at the first MTPJ have been reported as 8–10% [13, 14] although this increases with age [15], and affects almost half of the population over 80 years of age [16]. However, not all radiographic OA is symptomatic and a UK study of adults aged over 50 years reported a prevalence of symptomatic radiographic first MTPJ OA of 8% [5]. Treatment of first MTPJ OA varies, and ranges from conservative interventions of advice, information about the condition, and referral for surgery [17]. Recently updated guidance from the UK National Institute for Health and Care Excellence (NICE) emphasise the key role of non-pharmacological therapies in the management of OA [18]. NICE advocates intra-articular corticosteroid injections as an adjunct for short term pain relief to support exercise and weight loss, or when other pharmacological treatments are ineffective or unsuitable although none of these recommendations are specific to the first MTPJ [18, 19].

A recent UK trial demonstrated the effectiveness of intra-articular corticosteroid for hip OA to reduce pain and improve function. However, doubts remain about their effectiveness beyond the hip and knee, and possible chondrotoxic effects of recurrent injections have been noted [20, 21]. This uncertainty is reflected in the latest NICE guideline which emphasises the lack of consistent evidence on corticosteroids, particularly in joints other than the knee [18]. Both NICE and a recent James Lind Alliance Priority Setting Partnership have concluded that research is needed into the effectiveness of intra-articular corticosteroids for the management of OA in joints other than the knee, and particularly the foot and ankle [18, 22].

Different healthcare professionals treat people with first MTPJ OA; following changes in regulations for prescription only medicines in the UK, nurses and allied health professionals now prescribe and administer intra-articular corticosteroids [23]. Data demonstrate that intra-articular corticosteroids are used in the management of first MTPJ OA, but little else is known about this practice [17]. We aimed to explore current practice relating to the administration of intra-articular corticosteroids for people with painful first MTPJ OA amongst a range of UK health professionals working in the NHS.

## Methods

### Study design

We used a cross-sectional, self-administered, anonymous survey to elicit details of NHS clinical practice using the Qualtrics online survey platform (Qualtrics, Provo, UT, USA). Any healthcare professional administering intra-articular corticosteroid injections for painful first MTPJ OA within the NHS was eligible to complete the survey. Consent was implied by online completion, and the survey was accessible from November 2022 to January 2023. We report our findings in accordance with the Consensus-based checklist for Reporting of Survey Studies (CROSS) reporting guideline [24].

### Questionnaire design

Survey questions were drafted by experienced clinicians and foot and ankle researchers and then piloted with 18 healthcare practitioners from different professions and specialities including nursing, physiotherapy, podiatry, rheumatology, and orthopaedic surgery. The final survey comprised 16 questions that asked about respondents’ professional background, clinical service/setting, details of injection technique, equipment, injectate, timing and type of clinical follow up (Supplementary File 1).

We distributed the survey through professional bodies, special interest groups, and social media as outlined in Supplementary Table 1. We also targeted professional groups through social media (Twitter and Facebook), and personal and regional networks.

### Analysis

Survey data were analysed using SPSS v28 (Armonk, NY: IBM Corp). As this was an exploratory survey without pre-specified hypotheses, we present categorical data descriptively (*n* (%)) without inferential statistics. Due to the different legislation covering medical and non-medical professional prescribing, we present details of injectate separately between groups.

## Results

### Respondent characteristics

A total of 150 healthcare professionals completed the survey. Of these, six described their practice setting as private practice only, and were excluded from subsequent analysis, leaving 144 valid responses.

### Professional background and NHS settings

Respondents’ professional backgrounds and characteristics of the services in which they provided intra-articular corticosteroid injections varied (Table 1). Podiatrists were the most common profession responding to the survey (53/144; 39%), followed by orthopaedic surgeons (28/144;19%), podiatric surgeons (26/144; 17%) and physiotherapists (24/144; 16%). Most respondents were based in England (110/144; 76%). Although the majority of those injecting first MTPJs were based in dedicated foot and ankle services (96/144; 67%), many injections were delivered in other settings, including integrated musculoskeletal services, first contact practitioner clinics, physiotherapy clinics, and rheumatology services. These services were provided in hospital, community, and primary care settings.

**Table 1:** Professional and service characteristics of practitioners injecting 1st MTPJ

	***n*** **= 144 (%)**
**Profession**	
Podiatrist	53 (39)
Orthopaedic Surgeon	28 (19)
Podiatric Surgeon	26 (17)
Physiotherapist	24 (16)
Rheumatologist	2 (1)
Nurse	1 (1)
Surgical Care Practitioner	1 (1)
Not reported	9 (6)
**Professional group**	
Medical professional	31 (22)
Other healthcare professional	105 (73)
Not reported	8 (6)
**Nation**	
England	111 (77)
Scotland	7 (5)
Northern Ireland	3 (2)
Wales	3 (2)
Not reported	20 (14)
**Type of NHS service**	
Orthopaedic Foot & Ankle Surgery	39 (27)
Podiatry	30 (21)
Podiatric Surgery	28 (19)
Integrated Musculoskeletal	20 (14)
Physiotherapy	8 (6)
First Contact Practitioner	6 (4)
Rheumatology	4 (3)
Not reported	9 (6)
**Service Setting**	
Hospital	64 (44)
Community	38 (26)
Primary Care	11 (8)
Multiple (Hospital/Community and/or Primary care)	18 (13)
Intermediate Care	1 (1)
Not reported	12 (8)

### Injection delivery, equipment, and imaging

Half of respondents administered up to 25 injections per year (69/136; 49%) but some reported administering more than fifty corticosteroid injections per annum (21/136; 15%) (Table 2). Use of imaging to guide placement of injections varied. Although not using imaging guidance (i.e. anatomically guided) was the single most common response (52/136; 38%), nearly half of respondents routinely used either ultrasound or X-ray/fluoroscopy (65/136; 48%). Medical professionals were more likely to use imaging compared to non-medical professionals (21/31; 68% vs. 45/105; 43%). When imaging modalities are considered separately, it is clear that proportions of respondents using ultrasound are similar (medical professionals 10/31 (32%); non-medical professionals 35/105 (33%)) and that most of this difference is driven by higher use of x-ray/fluoroscopic guidance (medical professionals 11/31 (36%); non-medical professionals 9/105 (9%)).

**Table 2:** Volume of injections and use of imaging by professional background^a^

	Medical professional *n* = 31 (%)	Other healthcare professional *n* = 105 (%)	Total *n* = 136 (%)
**n. injections administered per year**			
1 to 25	22 (71)	45 (43)	67 (49)
26 to 50	5 (16)	26 (25)	31 (23)
51 to 100	1 (3)	11 (11)	12 (9)
> 100	1 (3)	8 (8)	9 (7)
Not reported	2 (6)	15 (14)	17 (13)
**n. injections (low/high)**			
Low (< 25)	22 (71)	45 (43)	67 (49)
High (26+)	7 (23)	45 (43)	52 (38)
Not reported	2 (6)	15 (14)	17 (13)
**Imaging guidance routinely used**			
No imaging used	8 (26)	44 (42)	52 (38)
Ultrasound	10 (32)	35 (33)	45 (33)
X-ray / Fluoroscopy	11 (36)	9 (9)	20 (15)
Tc99m bone scan	0 (0)	1 (1)	1 (1)
Not reported	2 (6)	16 (15)	18 (13)

^a^of those reporting profession (*n* = 136)

Equipment used and care pathways around injection treatment of first MTPJ OA varied (Table 3). The majority of respondents (*n* = 89, 62%) used a two-stage management pathway, inviting patients to attend a second appointment for injection after an initial assessment visit. Typical needle gauges used were 23 gauge (Blue) or 25 gauge (Orange) (81/136; 60%), although smaller 27 gauge needles were used by (15/136) 11% of respondents (Table 4).

**Table 3:** Clinical pathway

	***n*** **= 144 (%)**
**Typical clinical pathway**	
Assessed & injected at 1st outpatient appointment	26 (18)
**2nd appointment for injection**	
General outpatient clinic	44 (31)
Injection outpatient clinic	24 (17)
In theatre	17 (12)
Radiology outpatient	3 (2)
Day case procedure room	1 (1)
Not reported	29 (20)
**Typical follow-up**	
Routinely review all patients	68 (47)
Open appointment for return	32 (22)
No review	4 (3)
Not reported	40 (28)
**Timing of follow-up (if routinely review)**	***n*** **= 68 (%)**
1 month	14 (21)
2 months	18 (27)
3 months	22 (32)
6 + months	3 (4)
Not reported	11 (16)

**Table 4:** Injectate given by professional background

	Medical professional n (%)	Other healthcare professional n (%)	Total n (%)
**Preparation of steroid & LA**^**a**^	*n* = 31	*n* = 105	*n* = 136
Use premixed combination	1 (3)	41 (39)	42 (31)
Mix drugs in 1 syringe	22 (71)	18 (17)	40 (29)
Use 2 separate syringes	1 (3)	17 (16)	18 (13)
Do not use LA	3 (10)	10 (10)	13 (10)
Not reported	4 (13)	19 (18)	23 (17)
**Needle gauge used**	*n* = 31	*n* = 105	*n* = 136
21G (Green)	2 (6)	1 (1)	3 (2)
22G (Black)	2 (6)		2 (1)
23G (Blue)	15 (48)	36 (34)	51 (38)
25G (Orange)	6 (19)	24 (23)	30 (22)
27G (Grey)		15 (14)	15 (11)
29G (Red)		2 (2)	2 (1)
Not reported	6 (19)	27 (26)	33 (24)
**Use premixed methylprednisolone acetate + lidocaine hydrochloride**	*n* = 1	*n* = 41	*n* = 42
0.10 ml (4 mg)		24 (23)	24 (59)
0.20 ml (8 mg)		5 (5)	5 (12)
0.25 ml (10 mg)		7 (7)	7 (17)
0.70 ml (28 mg)		1 (1)	1 (2)
1 ml (40 mg)	1 (3)	2 (2)	3 (7)
Not reported		2 (2)	2 (4)
**Use non premixed steroids**	**n (%)**	**n (%)**	**n = 71**
Methylprednisolone acetate	*n* = 15	*n* = 22	*n* = 37
10 mg		1 (5)	1 (3)
15 mg		1 (5)	1 (3)
20 mg	1 (7)	2 (9)	3 (8)
30 mg		2 (9)	2 (5)
40 mg	10 (67)	11 (50)	21 (57)
80 mg	1 (7)	0 (0)	1 (3)
Dose not reported	3 (20)	5 (23)	8 (22)
Triamcinolone acetonide	*n* = 11	*n* = 22	*n* = 33
10 mg		5 (23)	5 (15)
20 mg		6 (27)	6 (18)
30 mg		1 (5)	1 (3)
40 mg	8 (73)	4 (18)	12 (36)
Dose not reported	3 (27)	6 (27)	9 (27)
**Use of non-premixed local anaesthetic**	*n* = 23	*n* = 35	*n* = 58
Lidocaine hydrochloride	8 (35)	15 (43)	23 (40)
Levobupivacaine hydrochloride	9 (39)	9 (26)	18 (31)
Bupivacaine hydrochloride	5 (22)	6 (17)	11 (19)
Mepivacaine hydrochloride		1 (3)	1 (2)
Not reported	1 (4)	4 (11)	6 (10)

^a^Premixed solution of methylprednisolone acetate + lidocaine hydrochloride. Doses for unmixed steroids captured as free-text

### Injectate administered

Methylprednisolone acetate and lidocaine hydrochloride were the most common steroid and local anaesthetic used in the injectate (Table 4). Notably, non-medical prescribers were more likely to use a premixed combination of the two drugs (41/105; 39%) compared to medical professionals (1/31; 3%). Three participants (all non-medical prescribers) reported using betamethasone sodium phosphate (2 mg, 40 mg and 80 mg). One medical professional reported using dexamethasone sodium phosphate (dose not reported).

There was a difference in steroid dose by professional background, with non-medical prescribers being more likely to administer lower steroid doses (4 mg of premixed methylprednisolone acetate and lidocaine hydrochloride was the most common response (24/105; 23%)) compared to medical prescribers (40 mg of methylprednisolone acetate was most common response (10/31; 32%)).

### Post-injection follow-up

The majority of respondents (*n* = 68; 47%) either reviewed all patients routinely or offered patients an open appointment following their injection (32/144; 22%). Most offering routine review follow-up patients within three months of their injection (54/68; 79%).

## Discussion

This is the first survey to explore the use and practice of intra-articular corticosteroid injections for symptomatic first MTPJ OA by UK health professionals. Intra-articular steroids are administered into the first MTPJ by a range of professionals across multiple NHS settings. We found large variation in practice across all stages of the procedure, including use of different drugs, doses, equipment, use of imaging, and follow-up care.

Treatment of first MPTJ OA is primarily driven by evidence from other joints which informs management guidelines. As a result, clinical practice varies, and ranges from advice, information and support, use of insoles, injections, through to surgery [17]. A 2022 systematic review of international clinical guidelines found considerable variation around their endorsement of intra-articular corticosteroids [25]. NICE clinical guidelines emphasise non-pharmacological interventions in the management of OA [26]. Recent changes now recommend that intra-articular corticosteroid injections are considered as a short-term adjunct to support therapeutic exercise or when other pharmacological treatments have failed. NICE also acknowledge the lack of evidence for corticosteroid injections for joints other than the knee [18]. There is a lack of evidence on the effectiveness of corticosteroid injections for first MTPJ OA.

Joint injections can be undertaken using anatomical (palpation) guidance or image-guidance. Our data show that use of imaging guidance is common when injecting the first MTPJ, and this included both ultrasound and x-ray/fluoroscopy. Ultrasound can be used in outpatient clinics, but x-ray/fluoroscopy is typically used in theatres so is likely to have considerable resource implications and could require patients to incur a period of time on waiting lists. In order to justify this additional cost and potential treatment delay, such imaging would need to improve clinical outcomes.

Inaccurate placement of joint injections has long been recognised in the literature with concerns that extra-articular placement may contribute to local tissue damage (soft tissue and fat atrophy) [27]. Whilst there is little evidence from the foot and ankle, image guidance has been shown to improve accuracy of injection placement in other joints but it is less clear whether it improves clinical outcome [28].

Within the first MTPJ, Heidari et al. (2013) [29] reported low rates of unintentional periarticular injection (i.e., missing the joint capsule) when using anatomical guidance to inject methylene blue into 106 cadaveric first MTPJs (10/106 joints; 9%). More recently, Reilly et al. (2022) [30] questioned the accuracy of this technique when injecting radio-opaque contrast in a cadaveric study (*n* = 6 feet). However, Razavi et al. (2021) [31] found no clinical benefit when using ultrasound guidance in a small trial of 50 people with first MTPJ OA randomised to landmark guidance or ultrasound guidance.

Similarly, Ekeberg et al. (2009) [32] found no difference in clinical outcomes when the same dose of triamcinolone acetonide (20 mg) was administered using either ultrasound guidance into subacromial bursa or an intramuscular injection into the gluteal region of people with rotator cuff disease. This suggests that accurate placement may not be important for symptom relief and raises questions on whether the additional cost of imaging is warranted. Our survey found that almost half of respondents used imaging guidance when injecting the first MTPJ with ultrasound and x-ray being the most common modalities. The use of imaging warrants further investigation.

Reilly (2021) recently highlighted the lack of a standardised protocol for injecting the first MTPJ, and proposed a useful framework for palpation-guided injections [33]. This protocol recommended using 23/25 gauge needles for injecting steroids and these were the most common sizes reported in our survey. Although a dose of 20 mg triamcinolone acetonide was recommended in the Reilly protocol, this contrasts with our findings, where more than twice as many respondents used methylprednisolone acetate compared to triamcinolone acetonide. Triamcinolone acetonide use was more common amongst medical prescribers than non-medical prescribers, and the most common dose was 40 mg.

Another key finding from our survey was the large variation in dose of each steroid injected. Generally, we found medical prescribers injected higher doses of steroid than non-medical prescribers but the variation between and within groups highlights the lack of evidence upon which to base clinical decisions. Methylprednisolone acetate was the most common steroid used, and the dose suggested in the Summary of Product Characteristics is 4-10 mg for small joints such as the metacarpophalangeal joints [34]. Although most respondents (36/42 86%) using the premixed solution of methylprednisolone acetate and lidocaine hydrochloride, (all non-medical prescribers), adhered to this suggested dose, only one respondent (also a non-medical prescriber) used this dose when using separate non-mixed steroid. Exploring justification for clinical practice was beyond the scope of this survey, current legislation prevents non-medical prescribers from combining drugs within a syringe prior to administration unless they have accreditation as an independent non-medical prescriber.

The most common dose of 40 mg methylprednisolone acetate is equivalent to 1ml, and most respondents administered 1ml or less of steroid. However, MRI data suggests the volume of synovial fluid within a healthy first MTPJ is much smaller than this, with a median (IQR) 0.15ml (0.073 to 0.21) [35]. Therefore, as well as the anti-inflammatory action of the steroid, the introduction of relatively large volumes of injectate will have a mechanical effect, potentially distending the joint capsule and distracting the joint. Which of these actions is more important in relieving symptoms may be worthy of further investigation.

A quarter of our survey respondents offered an open appointment for post-injection review or did not typically offer review, although one third failed to report their practice. Post injection review is essential to for monitoring of treatment response, which can guide future management, and identify any side effects which may require treatment. Effects of corticosteroids are widespread and although injection-related complications are rare, these can include post-injection flares, facial flushing, tendon and ligament rupture, subcutaneous fat atrophy, glucose tolerance impairment, osteonecrosis, osteoporosis, menstrual cycle irregularities, and skin pigmentation changes [36]. Few studies have reported adverse events after first MTPJ injections but adverse events may be acute or chronic with delayed onset, thus true incidence of complications is challenging to monitor [36]. There is uncertainty regarding the optimal method, timing, and clinical value of post-injection review.

In 2011, the American Orthopaedic Foot and Ankle Society (AOFAS) surveyed 197 of 870 registered members (23%) about their use of corticosteroid injections for a wider range of clinical conditions, but provided few data specific to first MTPJ OA [37]. With an average of 4.1 steroid injections per year, this suggests a much lower use of corticosteroid injections than our sample, but they also found methylprednisolone acetate and lidocaine hydrochloride were the most common injectate. Data from our survey is comparable to a survey of current practice in the care of carpometacarpal OA across 32 UK centres, showing a lack of clear guidance on the use of intraarticular steroid injection and uncertainty about their clinical effectiveness [38]. Injections were also administered into the carpometacarpal joint by a range of health professionals, using a mixture of anatomical and image guidance, with no standardisation in the threshold for injection. Similarly, no centre offered an injection at the first appointment, but the most commonly administered steroid and local anaesthetic was triamcinolone acetonide and lidocaine hydrochloride.

There are limitations to this survey, and perhaps most notable is the lack of sampling frame, thus it is unclear how representative these data are of UK practice. It is possible that orthopaedic surgeons are under-represented in our sample and we note that our respondents did not include radiologists. Another limitation is that due to the anonymous nature of the survey, we were unable to clarify extreme or unusual responses (e.g., use of Tc99m bone scan to guide injection placement). We did not explore respondent characteristics (gender, age, ethnicity, years of experience, qualifications), care pathways, nor use of concomitant therapies such as joint protection, exercise therapy, and manipulation under anaesthetic. However, this must be balanced against survey burden; we aimed to maximise response rates and minimise data missingness. Additional strengths include the piloting of our survey prior to use, reporting our findings in line with current recommendations, and wide distribution to encourage a spread of responses from different professionals across a range of healthcare settings.

## Conclusions

This is the first survey to investigate UK health professionals use of intra-articular corticosteroid for symptomatic first MTPJ OA. These injections are administered by a range of health professionals across the NHS. Methylprednisolone acetate and lidocaine hydrochloride were the most common steroid and local anaesthesia reported. However, there was large variation in clinical practice, including in the use of different corticosteroids, local anaesthetics, doses, equipment, use of imaging and care pathways. Despite the longstanding and widespread use of intraarticular injections for first MTPJ OA, there remains a lack of evidence to inform clinical decision making and this is reflected in the wide variation seen in practice.

### Supplementary Information

**Additional file 1. **Details of organisations distributing the online survey. 

## Data Availability

The datasets analysed during the current study are available from the corresponding author on reasonable request.

## Abbreviations

CROSS: Consensus-based checklist for Reporting of Survey Studies reporting guideline
MTPJ: Metatarsophalangeal joint
NHS: National Health Service
NICE: National Institute of Health and Care Excellence
OA: Osteoarthritis
