# Supplementary material for: A multi-professional survey of UK practice in the use of intra-articular corticosteroid injection for symptomatic first metatarsophalangeal joint osteoarthritis
Source: J Foot Ankle Res. 2023 Oct 17;16:71. doi: 10.1186/s13047-023-00672-6 (PMC10580568; doi:10.1186/s13047-023-00672-6)
Supplement: Supplementary file 1 — Additional file 1. Details of organisations distributing the online survey. [file 13047_2023_672_MOESM1_ESM.docx]

**Supplementary File 1.** Details of organisations distributing the online survey

| Organisation | Target Membership |
| --- | --- |
| Association of Foot & Ankle Physiotherapists (AFAP) | Foot and ankle physiotherapists |
| MSK:UK | Musculoskeletal podiatrists |
| Primary Care Rheumatology and Musculoskeletal Medicine Society (PRCMM) | GPs and AHPs with a special interest in MSK and rheumatology |
| Royal College of Podiatry | Podiatrists and Podiatric Surgeons |
| Social Media: Twitter | Any healthcare professional providing intraarticular corticosteroid injections for 1st MTPJ osteoarthritis within the NHS |
| Facebook | UK Podiatry Private Group: Podiatrists, Podiatry students and other AHPs to discuss any aspects of the podiatry profession. |
